# P-1792. Impact of Integrated Interventions of Prescriber Education and De-escalation Strategies on Implementing WHO Access: Watch Targets: Insights from a South Indian Tertiary Care Setting

**DOI:** 10.1093/ofid/ofae631.1955

**Published:** 2025-01-29

**Authors:** Jasper Victoria Leelarani Martinraj, Bibila Samuel, Feba Mary Thomas, Harini Senthil, Keren Ann George

**Affiliations:** C. L. Baid Metha College of Pharmacy, Chennai, Tamil Nadu, India; C. L. Baid Metha College of Pharmacy, Chennai, Tamil Nadu, India; C. L. Baid Metha College of Pharmacy, Chennai, Tamil Nadu, India; C. L. Baid Metha College of Pharmacy, Chennai, Tamil Nadu, India; C. L. Baid Metha College of Pharmacy, Chennai, Tamil Nadu, India

## Abstract

**Background:**

The study aims to determine the Access: Watch ratio, as recommended by the WHO AWaRE guide 2022, to regulate antimicrobial prescribing practices, highlighting the importance of educational sessions for successful AMS interventions.

**Table 1:**
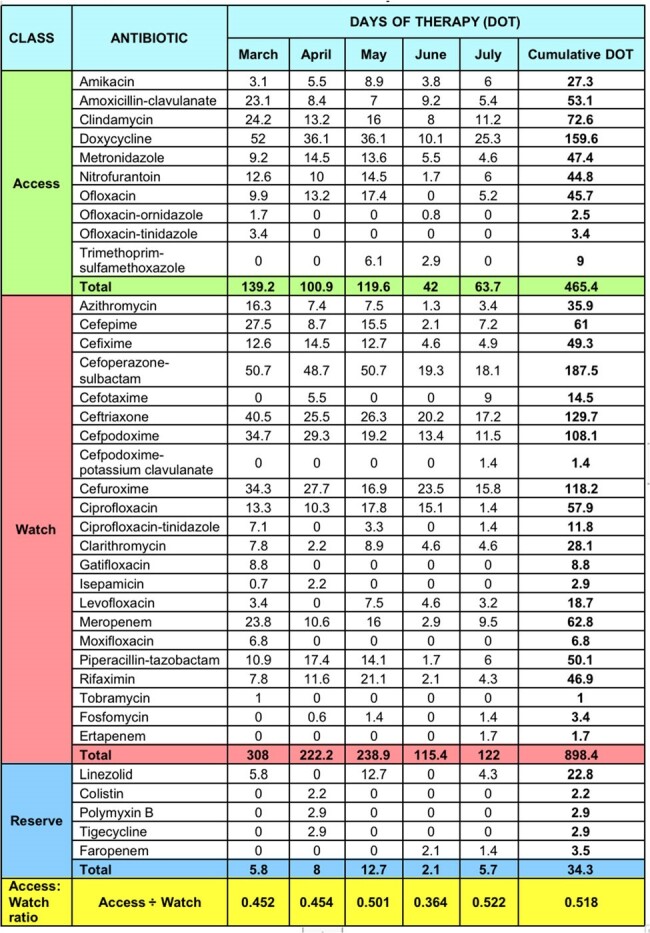
Quantification of antibiotic use during the study period Based on Days of therapy (DOT), an AMS metric.

**Methods:**

The study analyzed antimicrobial usage data from 360 patients from March to July 2023. It quantified DOT by comparing Access and Watch group antibiotics. The first three months focused on auditing antimicrobial prescribing practices, followed by a session on rational prescribing and an educational survey. The second two months focused on feedback on the impact of educational interventions.

**Figure 1:**
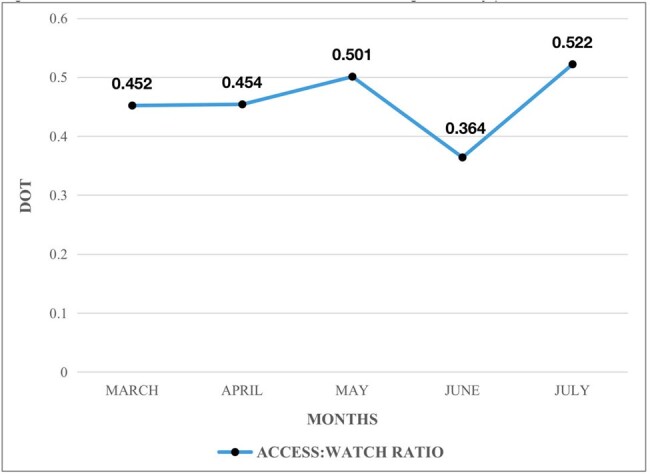
Trend of Access:Watch ratio of antibiotics during the study period

**Results:**

The use of Cefoperazone + Sulbactam, a broad-spectrum watch class antibiotic, was high in April and May (48.7 and 50.7, respectively) but decreased to 19.3 and 18.1 in the subsequent months following educational interventions. Optimization of de-escalation practices through audit and feedback strategies, emphasized in the continuing medical education sessions, contributed to this reduction. The Access: Watch Ratio increased from 0.45 to 0.52 over the study period, indicating a reduction in Watch antibiotic use. Pre-survey results showed 88.9% of prescribers considered de-escalation safer, increasing to 100% post-intervention. Various case-scenarios presented to prescribers showed improved understanding and adoption of rational antibiotic prescribing and de-escalation strategies after the educational session.

**Conclusion:**

Integrated interventions, including prescriber education and de-escalation strategies, were effective in achieving the WHO Access: Watch target in our South Indian tertiary care setting. The reduction in Watch antibiotic use and the increase in the Access: Watch ratio demonstrate the impact of these interventions on antimicrobial prescribing practices. Ongoing education and support for rational antibiotic use are critical in addressing antimicrobial resistance.

**Disclosures:**

**All Authors**: No reported disclosures

